# Boomerang Behaviour and Emerging Adulthood: Moving Back to the Parental Home and the Parental Neighbourhood in Sweden

**DOI:** 10.1007/s10680-020-09557-x

**Published:** 2020-03-18

**Authors:** Jenny Olofsson, Erika Sandow, Allan Findlay, Gunnar Malmberg

**Affiliations:** 1 Centre for Demographic and Ageing Research, Umeå University, 90187 Umeå, Sweden; 2 Department of Geography and Sustainable Development, University of St Andrews, St Andrews, UK; 3 Department of Geography, Umeå University, Umeå, Sweden

**Keywords:** Boomerang mobility, Life course, Young adults, Longitudinal, Returning home

## Abstract

This paper makes two original contributions to research on young adults’ boomerang mobility. First, it reveals the magnitude and complexity of return moves by young people to their parental home and neighbourhood. Secondly, it shows that the determinants and associates of return migration vary significantly when analysed at two different geographical scales—the parental home and the parental neighbourhood area. Using longitudinal data (1986–2009) on four cohorts of young adults, we find that boomeranging to the parental home in Sweden has increased in times of economic recession and is associated with economic vulnerability, such as leaving higher education or entering unemployment, and partnership dissolution. While returning to the parental home can offer financial support in times of life course reversal, we found gender differences indicating a greater independence among young women than men. Returning to the parental neighbourhood is found to be a very different kind of mobility than returning to co-reside with one’s parents, involving the migration decisions of more economically independent young adults. Results also indicate that returns to the parental neighbourhood, as well as returns to the parental home, can be part of young people’s life course changes.

## Introduction

Returning to the parental home is a significant mobility behaviour among young adults, and in the growing literature on this topic it has been claimed to be on the increase (Arundel and Ronald 2016; South and Lei 2015; Stone et al. 2014). Residential mobility research has focused on the return of young adults to the parental home due to concerns over the consequences this move might have on the child–parent relationship, the development of young people’s identities at a sensitive stage in their life course, and the implications for their subsequent relaunching into careers and becoming independent households once again (Sassler et al. 2008). Returning to the parental home has been seen as a reversal in the life course, a ‘failure’ transition triggered by, for instance, unemployment or divorce. It is also claimed that returning to the parental home—so-called boomeranging—becomes more common in times of increasing housing costs and insecure labour market conditions and is thus understood as a necessary solution to an unwanted situation (e.g. Arundel and Lennartz 2017; Albertini and Kohli 2012).

The conceptual significance of return mobility is threefold. First, it matters to those studying housing markets and so-called housing careers (Arundel and Ronald 2016). Second, it is important to demographers interested in the transition to adulthood and changing patterns of household composition and family ties (Billari and Liefbroer 2010). Third, it matters to researchers interested in the relationship between life courses and residential mobility trajectories (Coulter et al. 2016; Mulder and Wagner 2010; Davanzo and Goldscheider 1990). Mobility researchers have, however, been slow to draw attention to the importance of parallel return mobilities involving young people choosing to return to their parental neighbourhood rather than their parental home. While moving back to the parental home is seen as a reversal of the young adults’ housing career and a sign of their dependency on their parents, it is less clear to what extent returnees to the parental neighbourhood are in different or similar life situations.

Moving back to the parental neighbourhood can be stimulated by positive forces such as the increased well-being that flows from re-engaging in the social networks of the young adults’ wider society of upbringing, or by a desire for a higher quality of life than they have experienced during their initial period away from ‘home’ in order to study or complete an apprenticeship. It can be associated with a life course ‘success’, a transition by which young adults can capitalise on their investments in human capital. However, returning to the parental neighbourhood can also be a response to a situation of independence and a need for social and economic support from one’s family or wider social network, and a way to take advantage of the location-specific capital in one’s place of origin. Hence, returning to the parental neighbourhood may also be an unwanted but necessary life course reversal. It is therefore of conceptual interest to ask in what ways boomeranging to the parental neighbourhood replicates or diverges from return moves to the parental home.

This paper not only advances the research agenda on returns to the parental home, but also raises the question of whether a return move to the parental neighbourhood during emergent adulthood is of similar or different significance to returning to the parental home.

A longitudinal micro-dataset for the full population of four cohorts of young Swedes allows us to compare the drivers of boomerang behaviour on different geographical scales—those returning to the parental neighbourhood and those returning to the parental home. We analyse how turning points in the life course trajectory of young adults affect the risk of returning to the parental home. We also explore the extent to which these moves, in the context of the Swedish welfare regime, have been associated with situations of economic dependency and life course reversal, as has been found in the UK and the USA (Stone et al. 2014; South and Lei 2015). In addition, we examine the extent to which return moves to the parental neighbourhood are also associated with situations of economic and social independence.

### Why Do Young People Return?

Researchers have a longstanding fascination with how human mobilities have evolved over time in relation to changing demographic circumstances (Zelinsky 1971; Cooke 2011), and particularly in response to the uncertainties surrounding contemporary life courses. Cooke (2011) has hypothesised that a range of forces, including secular rootedness, mitigate in favour of a reduction in residential mobility over time. Others have noted the emergence of new mobilities associated with the increasingly individualistic nature of Western society, including higher levels of partnership dissolution, increased participation in higher education, and increased uncertainties associated with housing and labour market reversals (Champion and Shuttleworth 2016; Coulter et al. 2016). Return mobility by young people belongs to this set of new mobilities, and the term ‘boomeranging’ has been increasingly used to refer to the mobility of young people moving to and from their parental home (Kaplan 2009; Stone et al. 2014; Taylor et al. 2013).

The mobility of young people leaving their parental home has long been associated with key transitions involving education, entering work, marriage, and family formation (Elder 1977; Gee et al. 2003; Lundholm 2007; Nilsson and Strandh 1999). Demographers have noted that these transitions to adulthood have become ‘late, protracted and complex’ (Billari and Liefbroer 2010, 60). The complexities have included many life course ‘reversals’, stimulated by the termination of the very processes that may initially have encouraged young people to leave ‘home’. Thus, leaving education without secure employment as well as increased tendencies towards partnership dissolution (South and Lei 2015) appear to have triggered an increase in return moves by young people to their parental home. Returning to the parental home is often due to a constraint rather than a preference (Arundel and Lennartz 2017).

Young people’s economic situation is also often found to be one explanation for co-residing with their parents. Previous research has found that it is mainly a need of the adult–child that drives a return move, such as lack of economic resources due to unemployment, partnership dissolution, housing disruption, or childcare needs (Arundel and Lennartz 2017; Albertini and Kohli 2012; Sassler et al. 2008; Timonen et al. 2011).

In addition to changes in the economic and educational contexts within which young people leave the parental home, there have been other profound demographic and societal changes in the very nature of the life course. Arnett (2000), for example, has proposed ‘emergent adulthood’ as a new and distinct life course stage. He argues that it is not simply that this life course transition has come to be lengthened, but that emergent adulthood has become an identifiable life phase that is neither adolescence nor adulthood. It is ‘no longer normative for the late teens and early twenties to be a time of entering and settling into long-term adult roles’ (ibid, 469). Instead, the cultural construction of emerging adulthood has meant a delay in characteristics such as ‘accepting responsibility for oneself and making independent decisions’ (ibid, 473). It is in this context that residential mobility, including repeated periods of returning to the parental home, appears to have taken on a new significance in Western societies (Findlay et al. 2015; Coulter et al. 2016). Changes in the roles and identities of Western young people have also affected their residential behaviour in relation to their parents (Sassler et al. 2008), within the context of trends towards an increasingly individualistic society (Lesthaeghe 2010; Oppenheimer 2004).

Analysing different forms of return moves therefore involves studying the nature of the linked lives of young people and their parents (Bailey 2009; Elder et al. 2003). One of the first studies to use a large longitudinal dataset to examine young people’s linked lives and their boomerang behaviour was that by Stone et al. (2014). They hypothesised that boomeranging behaviour would increase with the passage of time as a result of the emergence of increasingly fluid life courses; on the one hand, it would be especially associated with the increasing number of young adults engaging in and leaving higher education (Champion and Shuttleworth 2016), and on the other it would also be triggered by partnership dissolution. Stone et al. confirmed that reverse turning points in an individual’s life course influenced the likelihood of young adults returning home, and found that gender and parenthood were critical in moderating mobility behaviour following partnership dissolution. In particular, they noted that in the UK men who experienced partnership dissolution and also had a child were more likely to return home (Stone et al. 2014).

The work by Stone et al. stimulated others to verify the extent of the boomerang phenomenon. Kleinepier et al. (2016), for example, found there to be cultural specificity in the roles assigned to young people, noting important differences between ethnic groups in their relationship with the parental home. In line with these ideas, Arundel and Ronald (2016) have asked whether more familialistic societies, such as those of southern Europe with their rather different welfare regimes, might experience different propensities in terms of boomerang behaviour. Several studies have shown that intergenerational co-residing is more common in the more familialistic societies of eastern and southern Europe than in northern Europe (e.g. Hank 2007; Fokkema and Liefbroer 2008). But as maintained by, for instance, Bengtson (2001) and Dykstra and Fokkema (2011), intergenerational family ties remain important, even in less familialistic countries. And several studies have demonstrated the role of family ties in adult children’s residential choice, for instance in the Netherlands (Smits 2010), as well as Sweden, with its strong welfare state (Pettersson and Malmberg 2009). However, in these two studies the focus was on older groups of adult children, compared to those examined in our study.

In a study of recently separated parents based on Swedish registers, Albertini et al. (2018) found that dissolution increased the likelihood of returning to their parental home, especially for men with low incomes who lived nearby. In a study from the USA, South and Lei (2015) found that returning to the parental home was associated with aspects such as family connectivity, physical victimisation, and poor parental health.

So far, empirical research has consistently examined boomerang behaviour in relation to young adults returning to their parental home. However, the complexity of the transition to adulthood (Billari and Liefbroer 2010) includes many other possible to-and-fro mobilities. A temporary ‘return home’ might not mean relocating to the parental home, but could also refer to the locality where one’s parents live or where one was raised.

Hjälm (2011), for instance, has noted that as parents age the younger generation may return to provide care, but that this type of boomeranging may not require co-residence. During emergent adulthood (as opposed to among people perhaps in their upper 30s or 40s), most boomerang moves to the parental neighbourhood are not likely to be stimulated by a need to care for the older generation, but rather by other factors. Moving to the parental neighbourhood can be interpreted to entail providing other support roles than the ones provided by those returning to live with their parents. Living near parents can involve help with childcare for young working parents, access to family members, a wider social network of friends and relatives, and other forms of location-specific capital (e.g. Mulder and Wagner 2012; Fischer et al. 1998) in the neighbourhood of origin. For many, these moves might be much like residential relocations to other places. For others, leaving higher education may simply involve an opportunity to engage in return migration to a desired residential neighbourhood that offers increased well-being across a spectrum of life domains (Nowok et al. 2013).

However, moving to an independent residence in the parental neighbourhood will inevitably require greater financial independence than would be the case among return movers who share accommodation with their parents. This is because returning to the parental neighbourhood incurs additional housing and living costs compared to returning to the parental home.

In this paper, we seize the opportunity to analyse the different geographical scales of boomerang behaviour, allowing for some theory-building by differentiating the benefits of returning to the parental home from those associated with the parental neighbourhood. Put in a different way, returning to the parental neighbourhood without co-residing with one’s parents may offer increased social support and a raised sense of well-being, as well as a delay in the independent decision-making associated with a full transition to adulthood (Arnett 2000). Clearly, boomerang behaviour of this kind will appeal to demographic groups rather different from those co-residing with their parents.

### The Swedish Context

An important contextual issue in research on boomerang mobility is the welfare model operating in different countries. In the more general Scandinavian or social democratic welfare model (Esping-Andersen 2013), there are better preconditions for young adults to have an independent housing career compared to many other European countries (Arundel and Ronald 2016). In Sweden, the availability of public housing facilitates independent housing and fewer returns to the parental home. Another important difference between Sweden and many other European countries is the extensive provision of public childcare and possibilities for parental leave (Viklund and Duvander 2017). Due to this, single or divorced Swedish parents, for instance, have better opportunities to manage without assistance from family members. Moreover, in Sweden, students from low-income backgrounds have relatively good possibilities to finance higher education, due to free tuition and more generous student loans (Weedon 2018). They are therefore more likely to be able to afford an independent residence. That said, however, it is important to note that in Sweden economic resources have become increasingly concentrated (Björklund et al. 2012) and housing costs are rising (Grundström and Molina 2016). Due to this, housing costs can become an obstacle to keep an independent residence after completing higher education when yet not established on the labour market. For less well-resourced young people, the option of returning to the parental home may therefore have become an increasingly popular post-study option.

Sweden, along with the other Nordic countries, has had the lowest rates of intergenerational co-residence compared to other European countries (Eurostat 2015). Intergenerational co-residence is a non-normative support strategy for independent living in Sweden and other Nordic countries. Here, financial support from parents to children is more common, whereas in southern Europe, for instance, co-residence is a more normative support strategy (Albertini and Kohli 2012; Hank 2007). As the options for financial support from parents may vary depending on the parents’ economic situation, this may affect young adults’ possibilities for independent living in contemporary Sweden.

### Hypotheses

While young Swedes exhibit a preference for independence and intergenerational co-residence in Sweden is still quite uncommon (Albertini and Kohli 2012), we examine how certain turning points in the life course trajectory in young adults’ life can trigger boomerang moves to the parental home more than others. We know that boomerang mobility is associated with partnership dissolution in Sweden (Albertini et al. 2018) and other countries (e.g. Stone et al. 2014; Smits 2010), and that there are gender differences in this mobility behaviour (ibid.). In this study we go further, analysing how other life events can trigger boomerang moves to the parental home. We also analyse whether some turning points in one’s life course are more or less associated with boomerang moves and whether there are gender differences in how these life-turning events relate to this mobility behaviour.

Firstly, we focus on how a dissolution in young adults’ life course is related to return migration to the parental home. While Albertini et al. (2018) studied boomerang moves to the parental home following dissolution among parents of all ages in Sweden, here we focus on young adults, both parents and non-parents. Previous research (e.g. Stone et al. 2014) shows that the gender and parenthood status of emergent adults operate selectively. International comparisons show that in Sweden the average age for nest-leaving is lower than that in most other European countries and that women leave their parental home at a younger age than men (Billari 2004; Angelini et al. 2011). We thus assume that young women are more likely to have an established independent residence than young men and are therefore less likely to move to their parental home after a dissolution (Hypothesis 1). Overall, women are more likely to stay with their children at the address previously occupied by the couple upon the dissolution of a partnership (e.g. Mulder and Wagner 2010; Mulder and Malmberg 2011). Mothers in Sweden, of all ages, have also been found to be less likely to move back to the parental home than fathers after a dissolution (Albertini et al. 2018). Thus, we assume that young mothers will be less likely to return to the parental home than young fathers following a dissolution (Hypothesis 2).

Secondly, as argued before, economic reversals in the life course are found to trigger return mobility by emergent adults to their parental home (e.g. Arundel and Lennartz 2017; Albertini and Kohli 2012). We therefore assume that young adults’ economic activity is associated with the likelihood of returning to live with their parents. As a consequence, we hypothesise that leaving full-time higher education and/or becoming unemployed will be associated with returning to the parental home (Hypothesis 3). While economic setbacks in young adults’ life course are assumed to increase the likelihood of returning to the parental home, the parents’ financial situation can also affect whether co-residence will be the solution. Co-residence with parents is more common among parents with lower economic resources as they have fewer opportunities to offer their child financial help, such as transfers for rent or a new home (Albertini and Kohli 2012; Gierveld et al. 1991; Grundy 2000). We therefore expect that young adults with high-income parents will be less likely to return home, since their parents will have better opportunities to provide them with financial support to sustain their independent lifestyle choices (Hypothesis 4).

Thirdly, we have argued that returning to the parental neighbourhood also provides support, but a different kind of support than the young people’s return migration to their parental home does. We expect that returning to the parental neighbourhood is not necessarily driven by life course reversals associated with partnership dissolution and other setbacks, but may be driven by young people having higher incomes and being able to afford their own accommodation and having residential preferences associated with living in their parental neighbourhood. We therefore hypothesise that returns by emergent adults to the parental neighbourhood will involve higher-income movers than boomerang moves to the parental home (Hypothesis 5).

Finally, a point of departure is that the possibilities for and constraints on returning to the parental neighbourhood differ from those involving returning to the parental home. This can generate differences in age and gender selectivity. As argued previously, returning to the parental neighbourhood can be associated with greater financial independence, achieved later during emergent adulthood and later in the life course compared to returning to the parental home. Therefore, we hypothesise that those returning to live in their parental neighbourhood are in the upper age range of 19–32 (Hypothesis 6). Regarding gender selectivity, as women usually establish themselves in the labour and housing market at younger ages compared to men, women can be expected to a greater extent to have achieved financial independence compared to men before age 32. We therefore hypothesise that women are more likely than men to return to the parental neighbourhood (Hypothesis 7).

## Methodology, Data, and Measures

### Data

The data used in this study originate from the Linnaeus Database (Malmberg et al. 2010), based on micro-data from various administrative registers provided by Statistics Sweden. The database contains anonymised individual records on all residents of Sweden, with annually updated information on demographic and socioeconomic characteristics such as gender, age, family status, education, occupation, income, coordinates of place of residence on a 100 m^2^ resolution, and multigenerational family relations. The longitudinal set-up of the data makes it possible to perform life course analyses of relations between events and various conditions over time with high-resolution data. In contrast to using census or panel data, these register data capture the entire population on a yearly basis, thus minimising the problems with, e.g. non-responses and measurement errors associated with many surveys.

### Population

We analysed boomerang mobility over time (1986–2009) for four cohorts of young Swedes. All 19-year-old Swedish residents in 1986, 1991, 1996, and 2001 (born in 1967, 1972, 1977, and 1982) who at that time point had moved from their parental home were followed until they (a) returned to the parental home or parental neighbourhood, (b) were lost to follow-up, (c) reached the age of 32, or (d) survived to the year 2009 (the last year of observation in the database).^1^ We have compared these groups with non-returners, as well as carried out analyses to examine differences between returners and other movers to test the samples in our analysis. The Swedish longitudinal sample encompasses 119,784 young people over 2591,060 time units (person-years) (see Table 1 for descriptive characteristics of these young people). Obviously, the group at risk of returning is a non-random selection, as demonstrated in previous research on nest-leaving (Nilsson and Strandh 1999). Hence, their propensity to return is influenced by various observable and non-observable characteristics, and they will have other migration propensities when compared to young adults in general.

**Table 1:** Characteristics of young adults in the sample (% of total person-years)

Variable	Category	% in each category		
		Female (*n* = 1 475 639 person-years)	Male (*n* = 1 115 427 person-years)	Total (*n* = 2 591 066 person-years)
Gender		56.9	43.2	100.0
Age group	19–24	46.4	46.2	46.3
	25–29	35.4	35.4	35.4
	30–32	18.3	18.4	18.4
Educational experience	Primary education	21.9	25.2	23.1
	Secondary education	55.2	52.9	54.3
	Post-secondary education	22.9	21.9	22.5
Individual income	Quartile 1	25.0	25.0	25.0
	Quartile 2	25.0	25.0	25.0
	Quartile 3	25.0	25.0	25.0
	Quartile 4	25.0	25.0	25.0
Country of birth	Sweden	48.5	62.6	54.6
	Outside Sweden	51.5	37.4	45.5
Change in economic activity	Student to employed	6.4	5.14	5.9
	Student to unemployed	1.9	1.74	1.8
	Unemployed to employed	5.2	5.1	5.1
	Employed to unemployed	5.1	3.9	4.6
	New student	5.7	3.9	5.0
	Stable student	17.1	13.6	15.8
	Stable unemployed	14.3	14.7	14.5
	Stable employed	44.4	52.1	47.3
Change in partnership status	New or stable partnered	28.3	19.9	25.2
	Dissolution	1.1	0.9	1.0
	Consistently unpartnered	70.6	79.2	73.8
Already a parent	Yes	34.4	16.7	26.8
*Parental income*				
Income mother	Quartile 1			25.2
	Quartile 2			25.9
	Quartile 3			25.5
	Quartile 4			23.3
Income father	Quartile 1			24.3
	Quartile 2			24.7
	Quartile 3			25.5
	Quartile 4			25.5

We follow young people from the age of 19 up to 32. Age 19 is chosen as this is the age of graduation from post-secondary education and an age at which most young adults in Sweden have moved from the parental home. The upper age limit of 32 means that 19-year-olds in 2001 had reached the age of 27 by 2009. Nevertheless, the analysis is based on a total population, representing all young people as the target of the analysis, rather than a sample.^2^ Furthermore, the long time period makes it possible to analyse possible changes in boomerang mobility behaviour over time. For these young people, we extracted information on several demographic and socioeconomic attributes from the database for the years 1986–2009, together with information on their mother’s and/or father’s place of residence and income.

### Measuring the Dependent Variables

As the data used are geo-referenced at a residential level, it is possible to examine how boomerang mobility behaviour can be understood at different geographical scales. We therefore scrutinise whether the analysed turning points in young adults’ life courses have the same relationship to returning to the parental home as to returning to the parental neighbourhood. Our analysis includes two dependent variables: the timing of returning to the parental home and the timing of returning to the parental neighbourhood.

Where one lives (home) is based on the place of residence at a 100-m^2^ resolution (100 × 100 m).^3^ We define living in their parental home as a young person living in the same 100-m^2^ as their father and/or mother. Returning to the parental home (boomeranging) is defined as the young person moving to the same 100-m^2^ as their mother and/or father at one point in time, and where their place of residence 1 year before (*t* − 1) was not in the same 100-m^2^. Since a small number of adult children may move to the same 100-m^2^ as their parent(s) without moving into the household, there is a minor overestimation of boomerang moves in the Swedish data. In our data, an individual’s place of residence is where they live in December of each year. This means that we can only know if someone has moved once a year. If residence spells in the parental home are for shorter periods (e.g. months), they will not be captured in this analysis. There could therefore also be a minor underestimation of non-registered moves by young people to their parental home. However, as you can only be registered at one residence at a time in Sweden, and since university students have strong incentives to register within the municipality where they study, this allows us to follow the vast majority of young people who have made a move from home. Moving closer to the parents is defined as the young adult moving to live within 5000 m of their mother and/or father, and where their place of residence 1 year before (*t* − 1) was more than 5000 m away from the parents. We define this 5000-m limit (5 km^2^) as the parental neighbourhood; it was chosen to include movers who end up within comfortable reach of their parents. Research has also shown that geographical distance is of importance for face-to-face contact, and that a distance of over 5 km makes a great difference in the nature of exchanges linked to social support (Knijn and Liefbroer 2006; Mulder and van der Meer 2009). To distinguish moving to the parental neighbourhood from boomeranging to the parental home, we have in the neighbourhood analysis excluded all young adults moving back to the parental home.

In this study, we focus only on moves by the young people towards their parents. While parents can also choose to move closer to their adult children, this has not been analysed in this study. Since the focus is on the circumstances of young people returning home or to the parental neighbourhood after nest-leaving, it is the first return move after the age of 19 that is in focus of this study. How long young people co-reside with their parents, or live in their neighbourhood, after a return move is therefore not analysed.

## Method

We use event history analysis (Allison 1982; Singer and Willett 1993) for our study of return moves and discrete-time logistic regression to model the probability of return migration to the parental home/neighbourhood at each point in time and as a function of turning points in the life course trajectory, and demographic and socioeconomic characteristics. To implement the event history analysis, we restructured the data into a person-year dataset. Thus, we examined the impact on boomerang moves of time-varying covariates, such as employment or family status from year to year, and events such as ending university studies or becoming unemployed.

The logistic regression model for the estimates of boomeranging by person i in the year *t* is:

$$\log [{\text{P}}_{\text{it}}/(1 - {\text{P}}_{\text{it}})] = \upalpha_{t} + {\beta} ^{\prime } {x}_{it}$$

where, α_*t*_ (*t* = 1, 2,…) is the constant term; *x*_*it*_ the explanatory variable; and *β* is the logistic regression coefficient.

The event (return migration) can only occur at discrete time points (years), and the transition from one discrete state to another can only occur once for each person; hence, only the first return migration is included in the analysis. Once the young adult has made a boomerang move, they are no longer at risk of moving again in that direction and are no longer observed.

All models were estimated separately for women and men to compare possible gender differences in determinants of boomerang mobility behaviour. First, we ran a basic model (Model 1) containing control variables representing the young adult’s individual socioeconomic and demographic characteristics, and then we added the variables representing the analysed turning points (Model 2) to test our hypothesis.

To ensure that our results relating to migration to the parental neighbourhood do not reflect overall migration behaviour by emergent adults, we undertook robustness checks of the models by testing different samples in the modelling. When we excluded the young people who moved somewhere else during the time of the study (other than to their parental home or neighbourhood), our results did not change (i.e. the same variables remained significant in the models when the reference group consisted of only emergent adults who did not move). Analysis was also undertaken based on a sample of those selecting other destinations than their parental neighbourhood (or parental home) and with non-moving young adults as a reference group.

To ensure that our results on return moves to the parental neighbourhood are not affected by the geographical distance from parents after nest-leaving, we also analysed a sample of young adults who had move at least 50 km from home. Although such a restriction gives a much smaller sample, the main results are relatively stable, indicating that returning to the parental neighbourhood is driven by the same factors regardless of the distance to the parents before the move.

### Life Course Turning Points

As argued above, return moves to the parental home are often driven by economic or social changes, so-called turning points, in the young adult’s life. Turning points, a central concept in the life course scholarship (Elder et al. 2003), refer to a significant transition in the individual’s life trajectory. Returning to the parental home is often brought about by a reversal in the individual’s life course trajectory, a ‘failure’ transition, such as becoming unemployed or going through a partnership dissolution (Davanzo and Goldscheider 1990). We largely followed the work of Stone et al. (2014) when creating variables indicating important turning points in Swedish young adults life course. The turning points in focus are related to change in economic activity and change in partnership status and are assumed to influence the risk of returning home. The economic activity each year captured whether the young person was employed, unemployed or inactive on the labour market, or a full-time student. Being employed was defined as having an annual income from work of at least 20,000 SEK (≈ 1872 EUR) at time t and not receiving a study allowance, to exclude those who are not part of the workforce (e.g. students). If the individual has received study allowance and their income from work was less than 20,000 SEK at time t, they were identified as a full-time student. A change in economic activity was categorised into eight categories: student to employed, student to unemployed, unemployed to employed, employed to unemployed, new student, stable student, stable unemployed, and stable employed (used as the reference category). A stable activity status refers to no change in activity between times t and *t* − 1. To capture possible effects of partnership dynamics on the risk of returning home, we defined the young adults’ partnership status each year as: new or stable partner,^4^ dissolution, and the reference category consistently unpartnered. A stable partnership (or being consistently unpartnered) refers to those with no change in marital status between times *t* and *t* − 1.

To control for how gender and parenthood may influence the relationship between partnership dissolution and returning home, we also measured the interaction effects between a change in partnership status and parenthood status. Being married and not having children was the reference category. The parenthood status of the young adults was defined as a dummy variable each year, where 1 represents the young adult being a parent at time *t* − 1. To more easily interpret these interaction effects, we calculated the predicted probabilities of returning home to one’s parent(s) using the coefficients from the regression models. We also calculated the predicted probabilities of returning home by change in economic activity. To capture any changes in boomerang mobility over time, we also controlled for interaction effects between time and age.

### Control Variables

Included in the analysis are also several time-varying covariates (Table 1), as well as fixed variables, including gender and being born in Sweden. The age variable was categorised into age groups for each year. The socioeconomic variables include educational experience, individual income, and parental income (mother and father, respectively) each year. Annual income from work was deflated according to the value of the Swedish krona in 2009. An individual’s income was used as an indicator of socioeconomic position for emergent adults and their parents. The variable was divided into four quartiles. Income information was available for the selected young adults as well as their parents. Educational experience was categorised into three levels based on the individual’s highest educational attainment each year (no post-compulsory education, post-compulsory education, and Bachelor’s degree or higher).

## Results

The starting point of this paper was the suggestion that an increasing share of young adults in contemporary Western societies engage in boomerang mobility. It has been suggested that returning to the parental home or neighbourhood may in part be a response to the changing contexts of life course transitions, and particularly to life course reversals. It has also been suggested that, in the case of returns to the parental neighbourhood, the protracted nature of emergent adulthood (Arnett 2000) may have contributed to a rise in new mobilities favouring semi-independent living close to but not at the same address as one’s parents.

### Boomerang to Parental Home

Our research shows that, in Sweden, 2.6% of the population aged 19–32 recorded a boomerang move to the parental home between 1986 and 2009. This can be compared to the UK, where the corresponding figure was 2.2% (Stone et al. 2014). While intergenerational co-residence is still rare in Sweden, our data confirm an increase in boomerang moves among young adults. Return migration was more frequent in the last period (2002–2009) compared to the first period (1986–1993). However, no linear trend towards increasing boomeranging was found (Table 3). Instead, there was a lower propensity to move back to the parental home in the second period, years that were characterised by economic stability compared to the first period, which included the crisis of the early 1990s. Similarly, the years 2002–2009 included the global economic crisis of 2008–2009. During this period, return moves increased once again. These findings reflect the notion that increased uncertainties on the housing and labour markets are associated with a rise in boomerang behaviour.

The pattern of boomeranging to the parental home varies by age and gender. As can be seen in Table 2, men aged 19–24 are the most likely to return and are significantly more likely to do so than women of the same age. While return rates drop with age, the gender differential remains (supporting Hypothesis 1). The regression coefficients for Model 1 (Table 3) confirm the downward gradient with age. Although interaction effects between age, period, and gender were complex, the dominant statistically significant trend in Model 2 is an increase in boomeranging by both genders in 2002–2009 relative to the period 1986–1993.

**Table 2:** Returning to parental home by age group and gender. 1986–2009 (Percentage of cohort with 95% confidence intervals)

Age group	Men	Women
19–24***	4.8 (4.7, 4.9)	3.5 (3.5, 3.6)
25–29***	2.2 (2.1, 2.3)	1.3 (1.3, 1.4)
30–32 ***	1.3 (1.2, 1.3)	0.7 (0.6, 0.7)
Total***	3.2 (3.2, 3.3)	2.2 (2.2, 2.3)

***p < 0.001 significant gender differences within age group. Significant gender differences for total sample. *N* = 28 559 person-years

**Table 3:** Coefficients from discrete-time logistic regression of young people returning to parental home, by gender

	Men		Women	
	Model 1	Model 2	Model 1	Model 2
*Period (ref. 1986–1993)*				
1994–2001	− 0.043	− 0.189***	− 0.006	− 0.149***
2002–2009	0.328***	0.295***	0.394***	0.305***
*Age group (ref. 19–24)*				
25–29	− 0.676***	− 0.583***	− 0.948***	− 0.653***
30–32	− 1.26***	− 1.36***	− 1.7***	− 1.32***
*Education (ref. Post-secondary education)*				
Secondary education	− 0.104***	− 0.121***	− 0.246***	− 0.144***
Primary education	− 0.032	− 0.038	− 0.244***	− 0.022
*Individual income (ref. Quartile 1)*				
Quartile 2	− 0.374***	− 0.376***	− 0.529***	− 0.507***
Quartile 3	− 0.706***	− 0.679***	− 0.848***	− 0.784***
Quartile 4	− 1.18***	− 1.1***	− 0.984***	− 0.863***
*Country of Birth (ref. Sweden)*				
Outside Sweden	− 0.386***	− 0.433***	− 0.33***	− 0.446***
*Income mother (ref. Quartile 1)*				
Quartile 2	0.099***	0.099**	0.115***	0.099***
Quartile 3	0.111***	0.109***	0.135***	0.111***
Quartile 4	0.085**	0.083**	0.199***	0.155***
*Income father (ref. Quartile 1)*				
Quartile 2	0.006	0.006	− 0.037	− 0.035
Quartile 3	0.034	0.036	− 0.028	− 0.036
Quartile 4	0.065*	0.069*	0.049	0.021
*Change in economic activity (ref. Stable employed)*				
Student to employed		0.128**		0.135***
Student to unemployed		0.275***		0.336***
Unemployed to employed		0.142**		− 0.043
Employed to unemployed		0.206***		0.068
New student		0.368***		0.443***
Stable student		− 0.041		0.046
Stable unemployed		− 0.052		0.061
*Change in partnership status (ref. New or stable partnership)*				
Dissolution		1.6***		1.99***
Consistently unpartnered		0.733***		0.995***
*Parent (ref. Non-parent)*				
Parent		0.055		− 0.299***
*Change in partnership status* *×* *parent (ref. New and stable partner and non-parent)*				
Dissolution × parent		0.048		− 0.407*
Consistently unpartnered × parent		− 0.126		− 0.463***
*Age group x Period (ref. 19–24 ×* *1986–1993)*				
25–29 × 1994–2001		0.053		0.078
25–29 × 2002–2009		− 0.226**		− 0.194**
30–32 × 1986–1993		(Empty)		(Empty)
30–32 × 1994–2001		0.554***		0.281***
30–32 × 2002–2009		(Omitted)		(Omitted)
Constant	− 2.19***	− 2.79***	− 2.45***	− 3.25***
Pseudo-R^2^	0.046	0.056	0.056	0.074

Table 3 also confirms that place of birth, income, education, and partnership status affect boomerang behaviour. Young adults born in Sweden were more likely to return to their parental home than those born abroad. It is also not surprising to note that those with the highest incomes were the least likely to return home, reflecting the greater economic independence of this group from their parents. A further income effect that the Swedish dataset lets us consider is the income of the parents of boomerang movers. We hypothesised that boomerang mobility was less likely among young adults with high-income parents (Hypothesis 4), as they could potentially offer their children financial support in times of economic setbacks, as an alternative to co-residence. Somewhat unexpectedly, the results reveal a positive correlation between mothers’ income and boomerang mobility (i.e. young men and women were more likely to return to their maternal home address if their mother had a high income), while for the paternal address the effect of paternal income was not significant. Finally, in introducing the effect of the control variables shown in Table 3 on the propensity to boomerang, it is worth noting that the effect of education is complex to interpret, but that those with a post-secondary education were more likely to return home than those without. This may reflect that those migrating for education are likely to return home upon completing their studies before taking up employment. This suggestion leads to our next arena of inquiry—that of the effect of life transitions on boomerang behaviour.

**Fig. 1:**
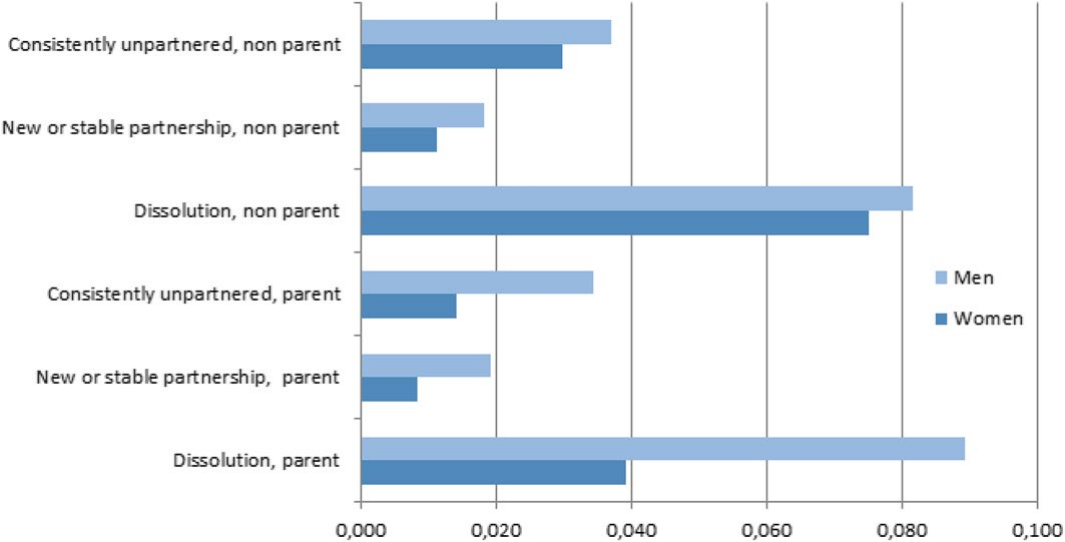
Predicted probability of returning home to parents by partnership status and parenthood status. All other covariates held constant

**Fig. 2:**
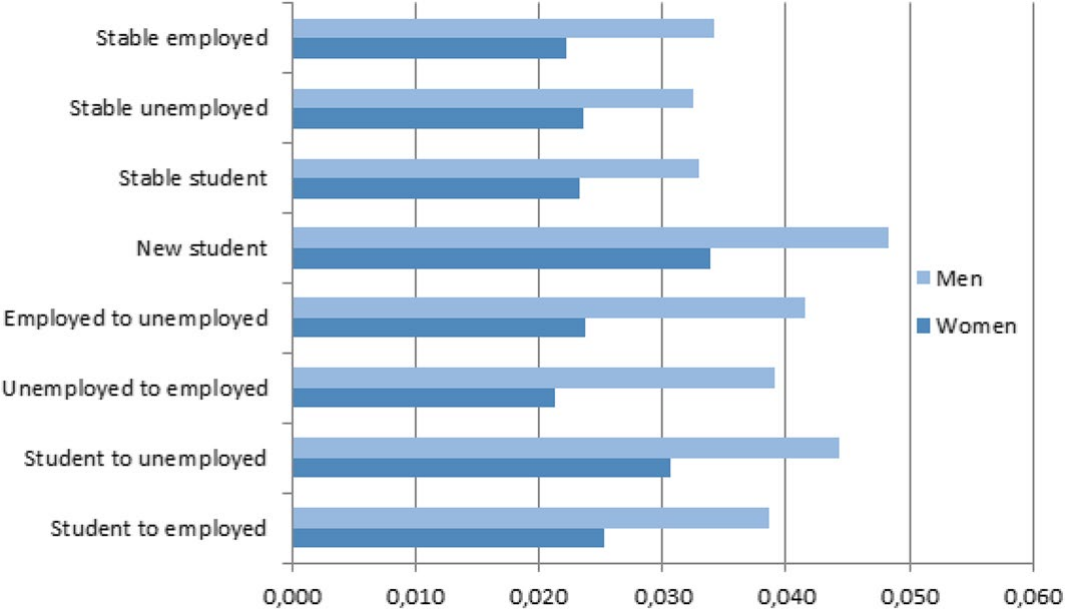
Predicted probability of returning home to parent by change in economic activity status. All other covariates held constant

Above all, the discrete-time regression allows us to address the paper’s hypotheses relating to turning points in the life course and the effects on return migration to the parental home. These transitions are shown graphically in Figs. 1 and 2, although the direction of the relationship is not graphed. Holding all other covariates constant, the Swedish data suggest that the likelihood of returning home among young Swedes is greatest following partnership dissolution. Figure 1 also supports Hypothesis 2 that after a partnership dissolution young mothers will be less likely to boomerang compared to young fathers.

Turning to changes in activity status as measured in Model 2 (Table 3), the results uphold the hypothesis that change, especially reversals in activity status, generates boomerang behaviour (Hypothesis 3). Consistent with Hypothesis 3, the calculated probabilities in Fig. 2 show that the greatest likelihood of returning to the parental home was found among new students and those entering unemployment. Table 3 confirms that, in comparison with those who remained employed, men and women who moved into unemployment or began studies were associated with an increased likelihood of returning home. This reflects that reversals in fortune led to the greatest increase in boomerang behaviour. Men were more likely than women to boomerang, regardless of economic transition. However, gender differentials in boomerang behaviour were the most striking for men entering unemployment and for men moving from unemployment into work.

### Returning to Parental Neighbourhood

We now turn to the case of return moves to the parental neighbourhood. When the return migration is to the parental neighbourhood (within 5 km), the age gradient (Table 4) is similar to that observed for returns to the parental home. The decrease with age is not as strong (compare with Table 2), which indicates that moving to live near one’s parents is more attractive for those over 25 years compared to those returning to co-reside with their parents. But still, we cannot confirm Hypothesis 6.

**Table 4:** Returning home to parental neighbourhood by age group and gender. 1986–2009 (Percentage of cohort with 95% confidence intervals)

Age Group	Men	Women
19–24***	3.0 (2.9, 3.1)	3.5 (3.4,3.6)
25–29**	1.8 (1.7, 1.9)	2.0 (1.9, 2.1)
30–32	1.4 (1.3, 1.5)	1.4 (1.3, 1.5)
Total***	2.3 (2.2, 2.3)	2.6 (2.5, 2.6)

***p < 0.001 **p < 0.05 significant gender differences within age group. Significant gender differences for total sample. *N* = 15,496 person-years

During this period, 2.5% of the population recorded a return move to the parental neighbourhood. In contrast to returns to the parental home, it appears that some different processes are at work here. Unlike those returning to the parental home, it is those with a higher income level (and thus greater financial independence) who return to their parental neighbourhood (Table 5). Moving back to the parental neighbourhood and having an independent residence may require a higher income. However, higher income levels do not inevitably correspond with the transition to adulthood and, for a proportion of these movers, may coexist with a desire to sustain some of the characteristics of emergent adulthood and the benefits of being close to one’s parents in order to avoid taking full responsibility for all the adult decision-making roles. As Table 5 shows, returning to the parental neighbourhood is also more likely among those with lower levels of education. However, consistent with Hypothesis 5, returning to the parental neighbourhood seems to result from migration decisions among young people who are economically independent of their parents, but who might have residential preferences or other motives which make their parents’ neighbourhood particularly attractive as a place of residence.

**Table 5:** Coefficients from discrete-time logistic regression of young people returning to parental neighbourhood, by gender

	Men		Women	
	Model 1	Model 2	Model 1	Model 2
*Period (ref. 1986–1993)*				
1994–2001	0.216***	− 0.388***	− 0.156***	− 0.335***
2002–2009	− 0.006	− 0.078	0.083**	0.025
*Age group (ref. 19–24)*				
25–29	− 0.506***	− 0.894***	− 0.551***	− 0.835***
30–32	− 0.716***	− 0.999***	− 0.954***	− 1.23***
*Education (ref. Post-secondary education)*				
Secondary education	0.446***	0.282***	0.289***	0.159***
Primary education	0.746***	0.52***	0.591***	0.404***
*Individual income (ref. Quartile 1)*				
Quartile 2	0.272***	0.278***	0.343***	0.315***
Quartile 3	0.481***	0.398***	0.399***	0.327***
Quartile 4	0.415***	0.309***	0.459***	0.365***
*Country of Birth (ref. Sweden)*				
Outside Sweden	− 0.122	− 0.117	0.126*	0.115
*Income mother (ref. Quartile 1)*				
Quartile 2	0.117*	0.127**	0.1***	0.105***
Quartile 3	0.052	0.067	0.11***	0.133***
Quartile 4	0.046	0.080	0.145***	0.185***
*Income father (ref. Quartile 1)*				
Quartile 2	− 0.084	− 0.076	0.036	0.045
Quartile 3	− 0.029	− 0.011	0.025	0.042
Quartile 4	− 0.044	0.001	− 0.013	0.02
*Change in economic activity (ref. Stable employed)*				
Student to employed		0.148*		0.177***
Student to unemployed		0.232		0.315***
Unemployed to employed		0.12		0.124*
Employed to unemployed		0.213*		0.224***
New student		− 0.088		− 0.014
Stable student		− 0.52***		− 0.363***
Stable unemployed		0.111		0.132**
*Change in partnership status (ref. New or stable partnership)*				
Dissolution		0.439		0.93***
Consistently unpartnered		− 0.127		− 0.068
Parent (ref. Non-parent)				
Parent		− 0.069		− 0.192**
*Change in partnership status × parent (ref. New and stable partner and non-parent)*				
Dissolution × parent		0.443		− 0.042
Consistently unpartnered × parent		0.35**		0.391***
*Age group x period (ref. 19–24* *×* *1986–1993)*				
25–29 × 1994–2001		0.393***		0.315***
25–29 × 2002–2009		0.186		0.078
30–32 × 1986–1993		(Empty)		(Empty)
30–32 × 1994–2001		0.29*		0.427***
30–32 × 2002–2009		(Omitted)		(Omitted)
Constant	− 4.06***	− 3.58***	− 4.13***	− 3.77***
Pseudo-R^2^	0.018	0.019	0.027	0.027

**Fig. 3:**
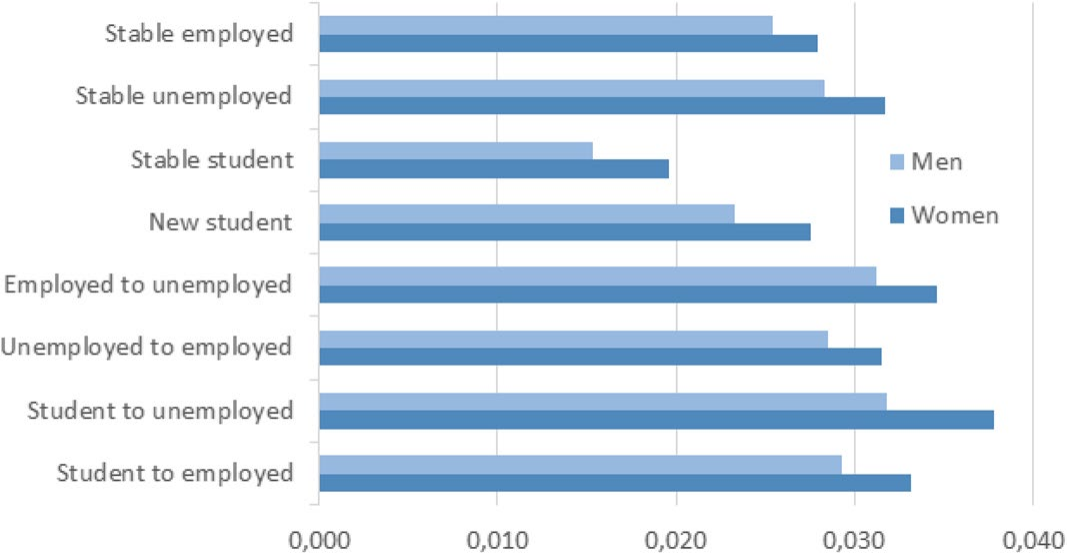
Predicted probability of returning to parental neighbourhood by change in economic activity status. All other covariates held constant

There are clear gender differences in migration behaviour when comparing return migration to the parental home and parental neighbourhood (Hypothesis 7). In contrast to the migration pattern for returning to the parental home, women are significantly more likely than men to return to the parental neighbourhood (Table 4). The results also reveal that this pattern increased significantly for women, but not for men, between 1986–1993 and 2002–2009 (Table 5). Unlike the migration pattern for returning to the parental home, whereby men were more likely to boomerang when their activity status changed, the predicted probabilities of returning to the parental neighbourhood area in Fig. 3 clearly show that women have higher probabilities of returning than men, regardless of economic activity status. There therefore seems to be a gender-selective effect in the attractions associated with being close to, but not living with, one’s parents. This is relatively easily explained for some groups, such as consistently unpartnered mothers seeing value in being close to their parents for advice and childcare support.

**Fig. 4:**
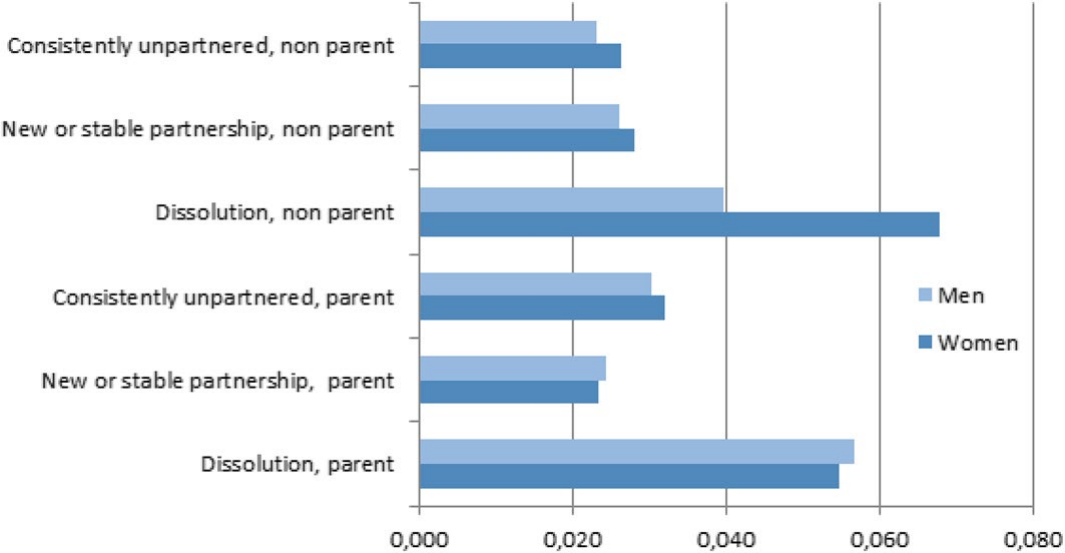
Predicted probability of returning home to parental neighbourhood by partnership status and parenthood status. All other covariates held constant

The results of Table 5 relating to partnership change contrast with those regarding return moves to the parental home (Table 3). It is only for young women experiencing partnership dissolution that returning to the parental neighbourhood significantly increases, compared to young women in a relationship. However, when examining the interaction effects between partnership status and parenthood with the calculated predicted probabilities (Fig. 4), we see that dissolution overall increases the chances of returning to the same area as the parental home, particularly for women with no children. Thus, having children does not in itself change the overall likelihood of moving to the parental neighbourhood. This result is in contrast to previous findings from the Netherlands on return migration by adult children over 30 (Smits 2010), and for Sweden on moving close to elderly parents (Pettersson and Malmberg 2009), since an association between moving close to parents and having children was found in both cases. These differences may be explained by the younger ages in our sample. Smits (2010), however, found an association between moving close to one’s parents and dissolution similar to what we found in our analysis (Fig. 4).

Since the reference group in our analyses included both stayers and movers to other places than the parental neighbourhood, additional regression analyses^5^ were carried out to check the robustness of our results by comparing the non-movers with movers to other places (i.e. moves that were neither a return to the parental neighbourhood nor a return to the parental home). For most of the variables, we found very similar results, indicating that the factors influencing moves to the parental neighbourhood are similar to those influencing moves to other places. However, the analyses revealed that, for moving elsewhere, the likelihood was significantly higher for men and women with higher education (i.e. opposite to the results in relation to boomeranging to the parental neighbourhood). One interpretation of this would be that, during emergent adulthood, those with lower education levels but with the means to live independently from their parents still find sharing their parental neighbourhood valuable in ways that go beyond childcare support and extend into other aspects of the distinct subjectivities and identities of this group, as outlined by Arnett (1997, 2000). In contrast, the labour market conditions for young adults with a high level of education trigger opportunities that encourage them to move farther from their parental home. As our research excludes any primary data collection on the identity formation of these young people, it is impossible to speculate any further on the direct effects of additional years of education in favouring a more rapid transition from emergent adulthood to fully independent adult identities.

Our robustness tests for exploring whether the geographical distance from the parents’ residential neighbourhood affects young people’s return move behaviour further strengthen our results. The results reveal that, irrespective of the distance from their parents, young people’s return moves to the parental neighbourhood are driven by similar turning points in the life course.

## Concluding Discussion

This paper has interrogated a large Swedish dataset to analyse ideas about the boomerang mobility of young adults following turning points in their life course trajectories. The paper makes an empirical contribution to the research literature on young people’s return migration, addressing returns of young adults in the context of a Scandinavian welfare state, using a large longitudinal micro-dataset and examining returning to both the parental home and neighbourhood. In addition, our aim is to chart the conceptual advance offered by our Swedish analysis. This is achieved through a theorisation of the distinctiveness of return moves to the parental neighbourhood compared with those to the parental home.

Although return moves to the parental home are still rare among Swedish young adults, the large dataset shows trends similar to those observed in other studies asserting that boomerang mobility is on the rise (Arundel and Ronald 2016; South and Lei 2015; Stone et al. 2014). The observed postponement of labour market entrance, family formation, and childbearing, as well as the extended period in higher education, are plausible explanations for the increase in boomerang mobility in Sweden. The transition to adulthood appears to be happening at a later age. Also in line with the research by Billari and Liefbroer (2010) and Arnett (2000), emergent adulthood has opened a longer time span for young people to engage in all the life activities and life course reversals that are possible for those in their late teens and early 20 s. Boomerang mobility to the parental home and return moves to the parental neighbourhood are forms of mobility that belong to this complex and changing part of the life course.

In this article, we find that the small but increasing share of young adults returning to live with their parents is driven by certain economic or social events shaping their life course trajectory. It is most likely that young adults in their early 20 s, particularly those completing higher education or becoming unemployed, will return to the parental home. Thus, in line with findings from the UK (Stone et al. 2014) and the USA (South and Lei 2015), the increased economic uncertainty of leaving higher education and entering unemployment had the expected effect on boomerang behaviour among young adults in Sweden (Hypothesis 3). Therefore, although our data do not convey the motives for return migration, it is reasonable to argue that for young adults who have achieved residential independence a return migration is likely to be driven by a constraint (Albertini et al. 2018; Arundel and Lennartz 2017). The logic that economic independence is likely to facilitate continuous residential independence is therefore supported. However, as neoliberal political agendas are advanced, in tandem with increasingly individualistic societal values, it seems entirely probable that this mobility response to economic uncertainty will grow in significance over time, while alternative provisions for young people in economically vulnerable positions (such as the provision of independent housing by a shrinking welfare state) will decline.

Gender differences were also confirmed, with greater independence observed among young women in relation to early nest-leaving and long-distance migration (Hypothesis 1). Overall, this may reflect a higher independence among young Swedish women. Our study also found that gender and parental status moderate the effect of partnership dissolution on Swedish young adults’ boomerang behaviour. As expected, partnership dissolution triggers young adults to return to the parental home. Although previous research on families with children in Sweden has highlighted how dissolution acts as a determinant for returning to live with one’s parents (Albertini et al. 2018), we can confirm here that dissolution increases the likelihood of boomerang moves even when non-parents are included in the analysis. We can also confirm that the effect was particularly marked for young fathers following their break-up with a partner (Hypothesis 2). Although shared custody is the common solution, women still take more responsibility for the children, and men to a larger extent move home to get assistance from their parents.

The high-quality income data available for the young adults’ parents allowed us to go beyond most prior studies on boomerang moves (e.g. Albertini et al. 2018; Arundel and Lennartz 2017; South and Lei 2015; Stone et al. 2014) and study the potential impact of the young adults’ mothers’ and fathers’ individual economic situation on return migration. Somewhat unexpectedly, young Swedish adults were significantly more likely to return to live with their mother if she was in the highest income quartile, while fathers’ income was not significantly associated with their children’s return-home mobility. Results regarding boomerang moves in the USA show that receiving financial help from family (based on family income) was not associated with young adults’ boomerang moves (South and Lei 2015). Non-economic factors, however, such as emotional closeness to one’s mother, were found to encourage young adults to return to their parental home (ibid.). Thus, our results may reflect the importance of non-observable factors in our study.

Our theorisation of boomerang mobility hypothesised that young people returning to their parental neighbourhood would differ from those returning to the parental home. In the absence of in-depth qualitative research, it is dangerous to make strong inferences as to the motives for returning to the parental neighbourhood, but the evidence that is available points to important similarities as well as differences. While returning to the parental home is interpreted as an unwanted move and as driven by an increasing lack of economic or social independency, returning to the parental neighbourhood without co-residing is a move to independent living, possible for young adults with a stronger economic position. Our results also indicate that young adults with financial resources are able to select their parental neighbourhood as a residential area and yet maintain residential independence, in line with Hypothesis 5. Several life course transitions related to economic activity were also strongly associated with boomeranging to the parental neighbourhood, once again especially for women. By moving to the parental neighbourhood, young adults may take advantage of a location-specific human capital (Mulder and Wagner 2012; Fischer et al. 1998) that they still have in the place where they were raised, including access to family members but also to a wider social network. However, our empirical analyses indicate that moving to the parental neighbourhood is more common for those who may need the support of a close social network, since we find associations with single parenthood and dissolution (for women). Thus, returning to the parental neighbourhood may also be driven by undesired events.

We could not confirm that returning to the parental neighbourhood is more likely among those in their early 30 s (Hypothesis 6). However, the moderating effect of gender (Hypothesis 7) and dissolution, together with the results regarding economic activity status and income level, leads to the conclusion that the support functions offered by returning to the parental home are very different from the opportunities offered by moving to the parental neighbourhood. Nonetheless, in situations and times of uncertainty, returning to the parental neighbourhood is attractive for many young adults even though it does not offer the fuller financial safety net of co-residence during periods of life course reversal.

In addition, our further analyses of residential mobility to other destinations revealed that there was a higher propensity for the highly educated to move elsewhere, and a higher rate of neighbourhood return for less well-educated groups. Further research is needed to establish whether it is the lack of high-skill jobs in the parental neighbourhood that explains the highly skilled preference for other destinations, or whether it is a positive preference by the less highly educated that explains their greater desire to return to the familiar environments proximate to their parental home.

While the analysis in this paper has made clear advances in extending existing work and suggesting new lines of research on the trend towards increased boomerang mobility among young adults, it is also evident that many issues remain unaddressed. Most notable of these is the need to test the robustness and significance of measurements of boomerang behaviour. So far, research has only taken account of movers in relation to an outward and return event. No investigation has considered the significance either of the length of the period of return, especially among those living with their parents, or of the sensitivity of the effects to the length of time spent away from their parents prior to return. A crucial topic for further research to explore involves the long-term outcomes of moving to the parental home versus neighbourhood, to determine the extent to which the return is only temporary, or a first step in settling close to one’s parents.

If the temporal duration of spells of absence and spells of return matters, then so too does the geography of the types of places selected for the initial move prior to return. For example, one might well ask with good reason, given the geographically differentiated housing and labour markets, whether moves in the Swedish context to the three metropolitan areas might be expected to generate higher or lower boomerang propensities, and whether return is more likely in the major cities with their very high housing costs than in smaller towns and rural areas (Hjälm 2011). Previous research has found that a larger proportion of young people living in densely populated regions have their parents close by, and that the distances between parents and adult children have decreased over time since the percentage of young adults born in the cities is larger today than in the 1990s (Kolk 2017; Malmberg and Pettersson 2007). Assuming that regional setting influences boomerang moves, the patterns of intergenerational proximity could affect the boomerang trends that are observed. Hence, there is a need for further research with analyses across different regional settings and of long- and short-distance moves, duration of co-residence, and the extent to which movers back to the parental neighbourhood are permanent returners.

This paper has suggested that return migration to the parental neighbourhood, as well as return migration to co-reside with parents, is an ongoing and significant phenomenon. For those with greater financial means, it appears to provide the potential for emotional and social support without the potentially harmful effects that co-residence can produce in terms of passivity among young people and the limitations placed on social networking (Sassler et al. 2008). On the one hand, the identification of return migration behaviour to the parental neighbourhood calls for a deeper theorisation of the phenomenon, while on the other it highlights the need to explore social policies for tackling the particular vulnerabilities of those with lower incomes who feel obligated to return to their parental home in the absence of other viable options, and who indeed feel *obligated* to return rather than selecting this move as a positive choice.

## Footnotes

1 This study is part of a financed project, with permission from Statistics Sweden to use data for the years 1986–2009. The individual-level dataset analysed in this study therefore only includes data up to 2009.

2 Young adults whose parents are both dead or live outside Sweden are not included in the analysis, as these individuals are not at risk of making a boomerang move or moving closer to their parents within Sweden.

3 Until 2011, it was not possible to define an individual's place of residence through register data in another way. From 2011, through the new Swedish dwelling register, it is possible to get information on all persons registered in the same household (Statistics Sweden 2013).

4 Having a partner refers to being married or having a registered partner. Cohabitation is not included in the analysis as the data available data do not provide information about cohabitation without children.

5 Details of the authors’ robustness tests are available on request but are not presented here due to space constraints.
